# Ethnoracial inclusion in clinical trials of psychedelics: a systematic review

**DOI:** 10.1016/j.eclinm.2024.102711

**Published:** 2024-07-03

**Authors:** Marcus E. Hughes, Albert Garcia-Romeu

**Affiliations:** a Department of Psychiatry, Yale School of Medicine, New Haven, CT, USA; b Interventional Psychiatry Service, Yale Psychiatric Hospital, New Haven, CT, USA; c Center for Psychedelic and Consciousness Research, Department of Psychiatry and Behavioral Sciences, Johns Hopkins University School of Medicine, Baltimore, MD, USA

**Keywords:** Psychedelics, Pharmacology, Diversity, Inclusion, Equity, Psychiatry

## Abstract

**Background:**

Prior data indicate limited ethnoracial diversity in studies testing psychedelic-assisted treatments. Regulatory approval for psychedelic treatments may be imminent given growing evidence for safety and efficacy in a variety of psychiatric conditions. Data on racial and ethnic inclusion rates in clinical psychedelic studies since 2018 have not been systematically reported to date. With the publication of multiple new studies in the field, an update to existing ethnoracial inclusion data is needed to inform the state of the science and future directions for research.

**Methods:**

Systematic review of Pubmed/MEDLINE, EMBASE, and Web of Science for studies of any design testing a psychedelic treatment for a psychiatric or substance use disorder published between January 1, 1994 and May 24, 2024. Search terms related to serotonergic psychedelics and MDMA, psychedelic therapies, psychiatric disorders, and substance use disorders were used. References of reviewed studies were screened for inclusion. Studies were rated for quality on a five-point scale ranging from 1 (most rigorous, i.e., properly powered randomized clinical trial) to 5 (least rigorous, e.g., case reports). Separate analyses were performed for two groups of studies, one involving all included studies meeting search criteria, and the other involving only studies from the USA. Rates of inclusion of different ethnoracial groups were calculated between studies published before and after December 31, 2017. Additionally, the proportion of White vs. non-White participants was compared between studies published before and after December 31, 2017. Finally, a nonparametric Mann–Whitney U test was used to compare the relative quality ratings of studies published before and after December 31, 2017.

**Findings:**

787 studies were screened, and 39 studies were included. This included 16 studies (n = 282) from a prior review published in 2018 with an additional 23 studies (n = 1111) that were published after 2017, consisting of 14 randomized controlled studies, 8 open-label studies, and 1 placebo-controlled, within-subject, fixed-order study. In all included studies published after 2017, 85.6% of participants identified as non-Hispanic White, 3.1% as Black, 6.8% as Latinx/Hispanic, 3.6% as Asian, 1.2% as Indigenous, 3.5% as mixed race, 1.4% as other, Pooled data from all included studies (n = 1393) found 85.0% of participants identified as non-Hispanic White, 2.9% as Black, 5.9% as Latinx/Hispanic, 3.2% as Asian, 1.9% as Indigenous, 3.7% as mixed race, 1.4% as other. In studies conducted in the USA (n = 1074), 908 (84.5%) of participants identified as White, 36 (3.4%) as Black, 80 (7.4%) as Latinx/Hispanic, 43 (4.0%) as Asian, 15 (1.4%) as Indigenous, 40 (3.7%) as Mixed, and 9 (0.8%) as Other. Differences in inclusion rates were found when comparing studies published before and after December 31, 2017 for all included studies and all studies conducted in the USA. The proportion of White to non-White participants was found to have decreased in studies conducted in the USA over the same period, but not for all included studies.

**Interpretation:**

Underrepresentation of ethnoracial minoritized populations persists in studies examining psychedelic therapies, despite growing calls for diversity. Non-Hispanic White participants remain an over-represented majority by a large margin, though, there were greater proportions of ethnic minoritized populations included in studies since 2018, particularly in studies conducted in the USA. This indicates progress towards equity in psychedelic research, though much work is needed to inform the safety and efficacy of psychedelic treatments in the general population.

**Funding:**

There was no funding source for this study.

**Research in context**

**Evidence before this study**

Psychiatric and substance-related conditions affect millions worldwide and contribute to morbidity and mortality across various populations. Many studies have examined psychedelic-assisted treatments for psychiatric and substance-related conditions yielding promising findings of efficacy and safety. There have been many published papers outlining the lack of ethnic and racial diversity in clinical research but only one paper, a 2018 review, which has been published outlining this disparity within psychedelic studies. EMBASE, Pubmed, and Web of Science databases were searched from January 1, 1994 through May 24, 2024 for terms related to various psychedelic drugs and psychiatric treatments. Studies of any design investigating a psychedelic drug in the treatment of a psychiatric condition were included if ethnoracial demographic data was collected. Studies of any language were included. The majority of studies were of moderate to high quality.

**Added value of this study**

This systematic review provides valuable insight into issues of diversity within clinical research involving psychedelic treatments and should act as a catalyst towards inclusive, equitable investigations of these therapies. The findings suggest non-Hispanic White people make up a great majority of participants in these trials which has been consistent over the past 30 years despite calls for increased diversity within clinical research.

**Implications of all the available evidence**

Limited participation of ethnic and racial minorities in psychedelic investigations hinders generalizability of results, which have been quite promising thus far. Not enough has been done to address issues of diversity, equity, and inclusion within this research despite the increasing number and scope of psychedelic clinical trials. To close the gap in diversity in clinical trials of psychedelic treatments, for both researchers and participants, thoughtful and targeted initiatives are needed.

## Introduction

The classic serotonergic psychedelics (e.g., psilocybin, lysergic acid diethylamide [LSD], N,N-dimethyltryptamine [DMT], 5-methoxy-N,N-dimethyltryptamine [5-MeO-DMT]) and 3,4-methylenedioxy-methamphetamine (MDMA) (hereafter, ‘psychedelics’) have historically been characterized as drugs of abuse.^1^ However, growing clinical research on these substances, often in conjunction with supportive therapy,^2^ suggests they may have broad therapeutic potential for a variety of psychiatric conditions.^3^ Psychedelic drugs can occasion profound alterations in consciousness and produce subjective experiences that may contribute to therapeutic effects as well as abuse potential.^4^ MDMA and psilocybin are currently considered “breakthrough therapies” by the U.S. Food and Drug Administration (FDA),^5^ a designation meant to expedite research on the basis of promising preliminary findings, and both were recently approved for medical use in Australia.^6^ FDA approval of MDMA-assisted therapy for post-traumatic stress disorder (PTSD) could occur as early as 2024.^7^ Data on psilocybin as a rapid-acting antidepressant may lead to future FDA approvals,^8^, ^9^, ^10^, ^11^, ^12^ and evidence regarding its therapeutic benefits for other conditions such as substance use disorders^13^^,^^14^ and existential distress is also accumulating.^15^, ^16^, ^17^, ^18^

Though the promise of novel therapies for PTSD, mood, and substance use disorders, among the most widespread and deleterious mental health conditions,^19^ would normally be cause for enthusiasm, concerns about the generalizability of available data and the representativeness of clinical trial samples have been raised.^20^^,^^21^ A 2018 review of 18 psychedelic clinical trials (n = 282) published between January 1, 1994 and December 31, 2017, found ethnoracial minority participation in modern psychedelic studies is low with non-Hispanic Whites representing a disproportionate majority, 82.3%, of study participants.^20^ This is problematic for several reasons, as clinical efficacy of psychedelic interventions and best practices in their delivery could vary in important ways across individuals of different cultural and ethnoracial backgrounds,^22^^,^^23^ and data on mental health disparities indicate an urgent need for effective mental health interventions among minoritized ethnoracial groups who have been underrepresented in modern psychedelic trials to date.^24^^,^^25^ The present review provides updated data on rates of inclusion of ethnoracial groups in psychedelic studies since 2018, and suggests actionable steps to increase ethnoracial diversity in these studies going forward.

## Methods

### Search strategy and selection criteria

**Fig. 1:**
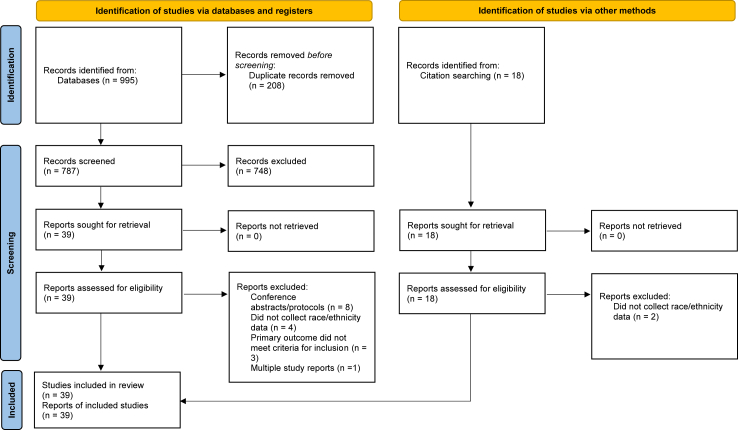
**PRISMA 2020 flow diagram for new systematic reviews which included searches of databases, registers and other sources**.^26^

Our protocol was not registered with PROSPERO as this database only registers systematic reviews with health-related outcomes. Ethics approval and informed consent were not required for this systematic review. We conducted this review in line with the PRISMA reporting guidelines.^26^ Pubmed/MEDLINE, EMBASE, and Web of Science were searched using the terms ((DMT) OR (5-MeO-DMT) OR (Ayahuasca) OR (dimethyltryptamine) OR (Psilocybin) OR (LSD) OR (MDMA) OR (lysergic acid diethylamide) OR (mescaline) OR (Ibogaine) OR (psychedelic)) AND ((treatment) OR (therapy)) AND ((randomized) OR (randomised) OR (quantitative) OR (pilot) OR (open label) OR (study) NOT (canna∗)). Both authors independently executed the search on May 24, 2024 and screened all studies for inclusion. This review included studies of any language and design using a psychedelic treatment for a psychiatric or substance use disorder. Studies were excluded if ethnoracial demographic data were not reported (Fig. 1). Study investigators were contacted to provide missing data if reported demographics were incomplete. Disagreements regarding study eligibility were resolved through discussion between the authors.

For more information, visit: http://www.prisma-statement.org/.

Consistent with a previous review,^20^ we included studies published after the 1993 National Institutes of Health (NIH) Revitalization Act which called for increased participation of minorities and women in funded-study populations,^27^ and focused specifically on studies of MDMA and serotonergic psychedelics (i.e., psilocybin, LSD, ibogaine, ayahuasca, and DMT) given recent increases in clinical research with these substances, their current legal status as highly restricted Schedule I compounds, and their potential approval for medical use in the foreseeable future. Although, many additional drugs can produce similar psychoactive effects to psychedelics and may also be used for therapeutic purposes (e.g., ketamine, nitrous oxide, cannabis), these are generally less restricted and not currently being considered for approval as new medicines. We therefore considered the examination of inclusion rates in studies evaluating these drugs as beyond the purview of this manuscript.

### Data analysis

Racial demographic data were compiled, and rates of participation were calculated using descriptive statistics. Because the only prior systematic review examining participant demographic data in clinical trials of MDMA and serotonergic psychedelics included studies published before December 31, 2017,^20^ we used this as a set comparison point to determine if a significant change in participant diversity had occurred since that time. We considered this the most appropriate analytic strategy in light of recent and substantial increases in clinical psychedelic research, as reflected by designation of four psychedelic substances as breakthrough therapies by the FDA since 2018. Rates of inclusion of different ethnoracial groups were compared between studies published before and after December 31, 2017. Additionally, the proportion of White vs. non-White participants were compared between studies published before and after December 31, 2017. Furthermore, in accordance with PRISMA guidelines,^26^ studies were rated for quality, by both authors, on a five-point scale ranging from 1 (most rigorous, i.e., properly powered randomized clinical trial) to 5 (least rigorous, e.g., case reports). Quality ratings' inter-rater reliability was calculated as an unweighted Cohen's kappa score using the web-based GraphPad QuickCalc tool. Meta-analysis and risk of bias calculation were not performed as we did not evaluate measured clinical outcomes but rather descriptive demographic data. In addition, selection bias by method of recruitment has not been found to significantly affect the diversity of participants in psychedelic studies.^20^ Finally, a nonparametric Mann–Whitney U test was used to compare the relative quality ratings of studies published before and after December 31, 2017.

Geographic and individual-level differences in ethnic and racial self-identity can obscure accurate demographic categorization of participants as race and ethnicity are constructs shaped and informed by societies on global and local levels, which may differ cross-culturally, and can change over time.^28^ This may be particularly true for countries outside of the USA with relative ethnic and racial population homogeneity. Participant race and ethnicity data presented here are based solely on self-report, regardless of where the studies were conducted. We included a category of “Latinx” to encompass all those who identify as Latin or Hispanic regardless of one's racial identity. However, for studies conducted in countries such as Spain or Mexico, where participant identification as Latinx or Hispanic may not have been explicitly assessed, we did not categorize these individuals as Latinx post-hoc, and report the ethnic and racial data as provided. To account for potential differences in demographic reporting internationally, separate analyses were performed for two groups of studies, one involving all included studies meeting search criteria, and the other involving only studies from the USA, which comprised the majority of included studies.

### Role of the funding source

There was no funding source for this study.

## Results

A summary of pooled results from included studies is provided (Table 1). 787 studies were screened, and 39 total studies were included. Included studies were conducted in various countries including Brazil (n = 1), Mexico (n = 1), New Zealand (n = 1), Switzerland (n = 4), Spain (n = 1), Canada (n = 1), the UK (n = 4), the Netherlands (n = 1), and the USA (n = 25). Regarding substance, these studies involved ayahuasca (n = 2), ibogaine (n = 2), LSD (n = 2), MDMA (n = 10), DMT (n = 1), 5-MeO-DMT (n = 1), 5-MeO-DMT and ibogaine (n = 1), and psilocybin (n = 20). Before 2018, 7 out of 16 studies (43.8%) were conducted primarily in the USA, accounting for 146 of 282 (51.8%) participants. Since 2018, 18 out of 23 studies (78.3%) were conducted primarily in the USA, comprising 928 of 1111 (83.5%) participants. Thus, most trials and participants overall were based in the USA.

**Table 1:** Pooled results of reported participant ethnoracial inclusion rates across clinical trials of psychedelics.

	Psychedelic	Country	N	White n (%)	Black n (%)	Latinx^e^ n (%)	Asian n (%)	Indigenous n (%)	Mixed n (%)	Other n (%)
**Studies published from 1993 to 2017**										
Moreno et al., 2006^29^	Psilocybin	USA	9	8 (88.9)	0 (0.0)	0 (0.0)	0 (0.0)	0 (0.0)	1 (11.1)	0 (0.0)
Bouso et al., 2008^30^	MDMA	Spain	6	6 (100.0)	0 (0.0)	0 (0.0)	0 (0.0)	0 (0.0)	0 (0.0)	0 (0.0)
Grob et al., 2011^18^	Psilocybin	USA	12	11 (91.7)	0 (0.0)	0 (0.0)	1 (8.3)	0 (0.0)	0 (0.0)	0 (0.0)
Mithoefer et al., 2011^31^	MDMA	USA	20	20 (100.0)	0 (0.0)	0 (0.0)	0 (0.0)	0 (0.0)	0 (0.0)	0 (0.0)
Thomas et al., 2013^32^	Ayahuasca	Canada	12	0 (0.0)	0 (0.0)	0 (0.0)	0 (0.0)	12 (100.0)	0 (0.0)	0 (0.0)
Oehen et al., 2013^33^	MDMA	Switzerland	14	12 (85.7)	0 (0.0)	0 (0.0)	1 (7.1)	0 (0.0)	1 (7.1)	0 (0.0)
Gasser et al., 2014^34^	LSD	Switzerland	11	11 (100.0)	0 (0.0)	0 (0.0)	0 (0.0)	0 (0.0)	0 (0.0)	0 (0.0)
Johnson et al., 2014^13^	Psilocybin	USA	15	14 (93.3)	0 (0.0)	0 (0.0)	1 (6.7)	0 (0.0)	0 (0.0)	0 (0.0)
Bogenschutz et al., 2015^35^	Psilocybin	USA	10	3 (30.0)	1 (10.0)	4 (40.0)	0 (0.0)	1 (10.0)	0 (0.0)	0 (0.0)
Griffiths et al., 2016^16^	Psilocybin	USA	51	48 (94.1)	2 (3.9)	0 (0.0)	1 (2.0)	0 (0.0)	0 (0.0)	0 (0.0)
Ross et al., 2016^17^	Psilocybin	USA	29	26 (89.7)	0 (0.0)	0 (0.0)	0 (0.0)	0 (0.0)	0 (0.0)	3 (10.3)
Carhart-Harris et al., 2016^36^	Psilocybin	UK	12	9 (75.0)	2 (16.7)	1 (8.3)	0 (0.0)	0 (0.0)	0 (0.0)	0 (0.0)
Brown & Alper, 2018^37^^,^^a^	Ibogaine	Mexico	30	27 (90.0)	0 (0.0)	1 (3.3)	0 (0.0)	0 (0.0)	0 (0.0)	2 (6.7)
Noller, Frampton & Yazar-Klosinski, 2018^38^^,^^a^	Ibogaine	New Zealand	14	14 (100.0)	0 (0.0)	0 (0.0)	0 (0.0)	0 (0.0)	0 (0.0)	0 (0.0)
Carhart-Harris et al., 2018^39^^,^^a^	Psilocybin	UK	8	6 (75.0)	1 (12.5)	0 (0.0)	1 (12.5)	0 (0.0)	0 (0.0)	0 (0.0)
Palhano-Fontes et al., 2019^40^^,^^a^	Ayahuasca	Brazil	29	17 (58.6)	1 (3.4)	0 (0.0)	0 (0.0)	0 (0.0)	11 (37.9)	0 (0.0)
*Studies from 1993 to 2017, N = 16*		*Total*	*282*	*232 (82.3)*	*7 (2.5)*	*6 (2.1)*	*5 (1.8)*	*13 (4.6)*	*13 (4.6)*	*5 (1.8)*
**Studies published from 2018**										
Ot'alora et al., 2018^41^	MDMA	USA	28	26 (92.9)	0 (0.0)	1 (3.6)	0 (0.0)	1 (3.6)	0 (0.0)	0 (0.0)
Mithoefer et al., 2018^42^^,^^b^	MDMA	USA	26	22 (84.6)	0 (0.0)	2 (7.7)	0 (0.0)	1 (3.8)	4 (15.4)	0 (0.0)
Danforth et al., 2018^43^	MDMA	USA	12	6 (50.0)	0 (0.0)	2 (16.7)	1 (8.3)	0 (0.0)	2 (16.7)	1 (8.3)
Wolfson et al., 2020^44^	MDMA	USA	18	15 (83.3)	1 (5.6)	0 (0.0)	0 (0.0)	1 (5.6)	0 (0.0)	1 (5.6)
Mitchell et al., 2021^45^^,^^c^	MDMA	USA	89	69 (77.5)	2 (2.2)	0 (0.0)	7 (7.9)	3 (3.4)	8 (9.0)	0 (0.0)
Sessa et al., 2021^46^	MDMA	UK	14	14 (100.0)	0 (0.0)	0 (0.0)	0 (0.0)	0 (0.0)	0 (0.0)	0 (0.0)
Davis et al., 2021^47^^,^^b^	Psilocybin	USA	24	22 (91.7)	1 (4.2)	0 (0.0)	1 (4.2)	0 (0.0)	0 (0.0)	0 (0.0)
Carhart-Harris et al., 2021^9^	Psilocybin	UK	59	52 (88.1)	0 (0.0)	0 (0.0)	0 (0.0)	0 (0.0)	0 (0.0)	7 (11.9)
Bogenshutz et al., 2022^14^^,^^d^	Psilocybin	USA	95	75 (78.9)	5 (5.3)	16 (16.8)	0 (0.0)	1 (1.1)	0 (0.0)	0 (0.0)
von Rotz et al., 2022^11^	Psilocybin	Switzerland	52	49 (94.2)	1 (1.9)	0 (0.0)	0 (0.0)	0 (0.0)	0 (0.0)	2 (3.8)
Goodwin et al., 2022^8^^,^^b^	Psilocybin (COMP360)	USA, CR, Denmark, Germany, Ireland, Netherlands, Portugal, Spain, UK, Canada	233	215 (92.3)	5 (2.1)	0 (0.0)	12 (5.2)	0 (0.0)	0 (0.0)	1 (0.4)
D'Souza et al., 2022^48^	DMT	USA	7	6 (85.7)	0 (0.0)	0 (0.0)	1 (14.3)	0 (0.0)	0 (0.0)	0 (0.0)
Holze et al., 2023^49^^,^^b^	LSD	Switzerland	42	42 (100.0)	0 (0.0)	0 (0.0)	0 (0.0)	0 (0.0)	0 (0.0)	0 (0.0)
Mitchell et al., 2023^50^	MDMA	USA	104	69 (66.3)	8 (7.7)	28 (26.9)	11 (10.6)	3 (2.9)	13 (12.5)	0 (0.0)
Sloshower et al., 2023^12^	Psilocybin	USA	19	16 (84.2)	2 (10.5)	0 (0.0)	0 (0.0)	0 (0.0)	1 (5.3)	0 (0.0)
Schneier et al., 2023^51^	Psilocybin	USA	12	9 (75.0)	0 (0.0)	0 (0.0)	3 (25.0)	0 (0.0)	0 (0.0)	0 (0.0)
Raison et al., 2023^52^^,^^c^	Psilocybin	USA	101	93 (92.1)	3 (3.0)	16 (15.8)	0 (0.0)	0 (0.0)	6 (5.9)	0 (0.0)
Goodwin et al., 2023^53^^,^^b^^,^^c^	Psilocybin (COMP360)	USA + Ireland	19	15 (78.9)	2 (10.5)	0 (0.0)	0 (0.0)	0 (0.0)	0 (0.0)	0 (0.0)
Peck et al., 2023^54^	Psilocybin	USA	10	9 (90.0)	0 (0.0)	1 (10.0)	0 (0.0)	0 (0.0)	0 (0.0)	0 (0.0)
Aaronson et al., 2023^55^^,^^c^	Psilocybin (COMP360)	USA	15	12 (80.0)	0 (0.0)	0 (0.0)	0 (0.0)	0 (0.0)	0 (0.0)	3 (20.0)
Reckweg et al., 2023^56^	5-MeO-DMT	Netherlands	16	16 (100.0)	0 (0.0)	0 (0.0)	0 (0.0)	0 (0.0)	0 (0.0)	0 (0.0)
Davis et al., 2023^57^	5-MeO-DMT + Ibogaine	USA	86	75 (87.2)	1 (1.2)	9 (10.5)	2 (2.3)	3 (3.5)	5 (5.8)	0 (0.0)
Agrawal et al., 2024^15^	Psilocybin (COMP360)	USA	30	24 (80.0)	3 (10.0)	1 (3.3)	2 (6.7)	0 (0.0)	0 (0.0)	0 (0.0)
*Studies since 2018, N = 23*		*Total*	*1111*	*951* (85.6)	*34 (3.1)*	*76 (*6.8)	*40 (3.6)*	*13 (1.2)*	*39 (3.5)*	*15 (1.4)*
*Pooled studies, N = 39*			*1393*	*1183* (85.0)	*41 (2.9)*	*82* (5.9)	*45 (3.2)*	*26 (1.9)*	*52 (3.7)*	*20 (1.4)*

Abbreviations: MDMA, 3,4-methylenedioxy-methamphetamine; DMT, N,N-Dimethyltryptamine; 5-MeO-DMT, 5-methoxy-N, N-dimethyltryptamine; USA, United States of America; CR, Czech Republic; UK, United Kingdom.

Note: We included from Carhart-Harris et al., 2018 only patients not already included in Carhart-Harris et al., 2016.

a Finalized pre-print data was included in previous review despite final publication after 2017.

b Authors were contacted to provide missing data.

c Paper contained incomplete ethnoracial demographic data.

d Sum greater than 100% as some participants selected multiple categories rather than identifying as “mixed”.

e Includes individuals identifying ethnically as Latinx or Hispanic regardless of the selected race category.

The previous review included preprint data from three papers which were published after 2017.^37^^,^^38^^,^^40^ These data were compiled with the pre-2018 group during statistical analyses.

Total sample size included in a previous review was n = 282, with 232 (82.3%) White, 7 (2.5%) Black, 6 (2.1%) Hispanic/Latinx, 5 (1.8%) Asian, 13 (4.6%) indigenous, 13 (4.6%) mixed race, and 5 (1.8%) other.^20^ Total sample size in studies published since January 1, 2018 was n = 1111, with 951 (85.6%) White, 34 (3.1%) Black, 76 (6.8%) Latinx/Hispanic, 40 (3.6%) Asian, 13 (1.2%) Indigenous, 39 (3.5%) mixed race, and 15 (1.4%) other. It should be noted that one study^14^ allowed participants to choose more than one ethnoracial category and two participants in that study chose two categories each. Three studies^45^^,^^52^^,^^53^ did not report ethnoracial demographic data for all study participants; patients without known race or ethnicity (n = 6) were excluded from statistical analyses. Inclusion of these data would not significantly alter our findings.

Pooled data from all included studies comprised a total sample of n = 1393, with 1183 (85.0%) White, 41 (2.9%) Black, 82 (5.9%) Latinx/Hispanic, 45 (3.2%) Asian, 26 (1.9%) Indigenous, 52 (3.7%) mixed race, and 20 (1.4%) other.

Comparisons of all included studies found differences in ethnoracial inclusion between studies published before and after December 31, 2017. Earlier studies had smaller proportions of Hispanic/Latinx, and Asian participants, and greater proportions of indigenous participants. Additionally, comparisons of proportions of White vs. non-White participants between studies published before and after December 31, 2017 found no substantial differences.

Quality ratings showed excellent inter-rater reliability with 37 of 39 (94.9%) ratings in agreement and an unweighted Cohen's Kappa of 0.924 (SE = 00.052). Quality ratings of all included studies (Table 2) found that overall, the studies published before 2018 were of lower quality with a median rating of 3 (i.e., retrospective cohort studies) compared to studies published since 2018, which had a median rating of 2 (i.e., well-designed controlled trial without randomization; prospective comparative cohort trial). However, this finding did not reach statistical significance (U = 129, p = 0.099.).

**Table 2:** Rating of quality of evidence of studies.

	Indication	Psychedelic	Study design	Quality of evidence^b^
**Studies published from 1993 to 2017 (N = 16)**				
Moreno et al., 2006^29^	OCD	Psilocybin	Open label dose–response	3
Bouso et al., 2008^30^	PTSD	MDMA	Double-blind, ascending dose study	4
Grob et al., 2011^18^	anxiety in advanced stage cancer	Psilocybin	Randomized, double-blind, active placebo controlled, cross-over trial	2
Mithoefer et al., 2011^31^	PTSD	MDMA	Randomized, double-blind, placebo controlled with open label crossover	2
Thomas et al., 2013^32^	addiction/SUD	Ayahuasca	Open label observational	3
Oehen et al., 2013^33^	PTSD	MDMA	Randomized, double-blind, active-placebo controlled trial	2
Gasser et al., 2014^34^	Anxiety associated with life threatening disease	LSD	Double-blind, randomized, active placebo-controlled with open-label crossover	2
Johnson et al., 2014^13^	Tobacco use disorder	Psilocybin	Open label pilot	3
Bogenschutz et al., 2015^35^	AUD	Psilocybin	Open label pilot	3
Griffiths et al., 2016^16^	Cancer-related distress	Psilocybin	Randomized, double-blind, active placebo controlled, cross-over trial	2
Ross et al., 2016^17^	Cancer-related distress	Psilocybin	Double-blind, placebo-controlled, crossover trial	2
Carhart-Harris et al., 2016^36^	TRD	Psilocybin	Fixed dose, open label	3
Brown & Alper, 2018^37^^,^^a^	OUD	Ibogaine	Open label, observational	3
Noller, Frampton & Yazar-Klosinski, 2018^38^^,^^a^	Opioid dependence	Ibogaine	Open label, observational	4
Carhart-Harris et al., 2018^39^^,^^a^	TRD	Psilocybin	Fixed dose, open label	3
Palhano-Fontes et al., 2019^40^^,^^a^	MDD	Ayahuasca	Double-blind randomized placebo-controlled trial	1
**Studies published from 2018 (N** = **22)**				
Ot'alora et al., 2018^41^	PTSD	MDMA	Randomized double-blind dose response comparison	2
Mithoefer et al., 2018^42^	PTSD	MDMA	Randomized, double-blind, dose–response, phase 2 trial	2
Danforth et al., 2018^43^	Social anxiety in ASD	MDMA	Randomized, double-blind, placebo controlled	2
Wolfson et al., 2020^44^	Anxiety related to life threatening illness	MDMA	Randomized, double-blind, placebo controlled with open label crossover	2
Mitchell et al., 2021^45^	PTSD	MDMA	Randomized, double-blind, placebo controlled	1
Sessa et al., 2021^46^	AUD	MDMA	Fixed dose, open label	3
Davis et al., 2021^47^	MDD	Psilocybin	Randomized, wait-list controlled	2
Carhart-Harris et al., 2021^9^	MDD	Psilocybin	Randomized, double-blind, controlled (comparative efficacy)	1
Bogenshutz et al., 2022^14^	AUD	Psilocybin	Randomized, double-blind, active placebo controlled	1
von Rotz et al., 2022^11^	MDD	Psilocybin	Randomized, double-blind, placebo controlled	1
Goodwin et al., 2022^8^	TRD	Psilocybin (COMP360)	Randomized, double-blind, controlled (dose comparison)	1
D'Souza et al., 2022^48^	MDD	DMT	Open label, fixed order, dose escalation	3
Holze et al., 2023^49^	Anxiety with and without life threatening illness	LSD	Randomized, double-blind, placebo-controlled, 2-period, random-order, crossover design	1
Mitchell et al., 2023^50^	PTSD	MDMA	Randomized, double-blind, placebo controlled	1
Sloshower et al., 2023^12^	MDD	Psilocybin	Placebo-controlled, within-subject, fixed-order study	3
Schneier et al., 2023^51^	Body dysmorphic disorder	Psilocybin	Open label	3
Raison et al., 2023^52^	MDD	Psilocybin	Randomized, double-blind, active placebo controlled	1
Goodwin et al., 2023^53^	MDD	Psilocybin (COMP360)	Fixed dose, open label	3
Peck et al., 2023^54^	Anorexia Nervosa	Psilocybin	Fixed dose, open label	3
Aaronson et al., 2023^55^	Bipolar II depression	Psilocybin (COMP360)	Fixed dose, open label	3
Reckweg et al., 2023^56^	TRD	5-MeO-DMT	Open label, dose escalation	3
Davis et al., 2023^57^	PTSD, Depression, Anxiety	5-MeO-DMT and Ibogaine	Open label, observational	3
Agrawal et al., 2024^15^	MDD in patients with cancer	Psilocybin	Fixed dose, open label	3

Abbreviations: AUD, alcohol use disorder; MDD, Major depressive disorder; PTSD, post-traumatic stress disorder; TRD, treatment resistant depression; OUD, opioid use disorder; ASD, autism spectrum disorder; OCD, obsessive compulsive disorder.

a Finalized pre-print data was included in previous review despite publication after 2017.

b Quality Rating Scheme: 1 = Properly powered and conducted randomized clinical trial; systematic review with meta-analysis; 2 = Well-designed controlled trial without randomization; prospective comparative cohort trial; 3 = Case-control studies; retrospective cohort study; 4 = Case series with or without intervention; cross-sectional study; 5 = Opinion of respected authorities; case reports.

A summary of pooled data from included studies conducted in the USA is also provided (Table 3), representing a sample of n = 1,074, with 908 (84.5%) White, 36 (3.4%) Black, 80 (7.4%) Latinx/Hispanic, 43 (4.0%) Asian, 15 (1.4%) Indigenous, 40 (3.7%) Mixed, and 9 (0.8%) Other.

**Table 3:** Pooled results of reported participant ethnoracial inclusion rates across clinical trials of psychedelics in the USA.

	Psychedelic	N	White n (%)	Black n (%)	Latinx^d^ n (%)	Asian n (%)	Indigenous n (%)	Mixed n (%)	Other n (%)	Quality of evidence^e^
**Studies published from 1993 to 2017**										
Moreno et al., 2006^29^	Psilocybin	9	8 (88.9)	0 (0.0)	0 (0.0)	0 (0.0)	0 (0.0)	1 (11.1)	0 (0.0)	3
Grob et al., 2011^18^	Psilocybin	12	11 (91.7)	0 (0.0)	0 (0.0)	1 (8.3)	0 (0.0)	0 (0.0)	0 (0.0)	2
Mithoefer et al., 2011^31^	MDMA	20	20 (100.0)	0 (0.0)	0 (0.0)	0 (0.0)	0 (0.0)	0 (0.0)	0 (0.0)	2
Johnson et al., 2014^13^	Psilocybin	15	14 (93.3)	0 (0.0)	0 (0.0)	1 (6.7)	0 (0.0)	0 (0.0)	0 (0.0)	3
Bogenschutz et al., 2015^35^	Psilocybin	10	3 (30.0)	1 (10.0)	4 (40.0)	0 (0.0)	2 (10.0)	0 (0.0)	0 (0.0)	3
Griffiths et al., 2016^16^	Psilocybin	51	48 (94.1)	2 (3.9)	0 (0.0)	1 (2.0)	0 (0.0)	0 (0.0)	0 (0.0)	2
Ross et al., 2016^17^	Psilocybin	29	26 (89.7)	0 (0.0)	0 (0.0)	0 (0.0)	0 (0.0)	0 (0.0)	3 (10.3)	2
*Studies from 1993 to 2017, N = 7*	*Total*	*146*	*130 (89.0)*	*3 (2.1)*	*4 (2.7)*	*3 (2.1)*	*2 (1.4)*	*1 (0.7)*	*3 (2.1)*	
**Studies published after 2017**										
Ot'alora et al., 2018^41^	MDMA	28	26 (92.9)	0 (0.0)	1 (3.6)	0 (0.0)	1 (3.6)	0 (0.0)	0 (0.0)	2
Mithoefer et al., 2018^42^^,^^a^	MDMA	26	22 (84.6)	0 (0.0)	2 (7.7)	0 (0.0)	1 (3.8)	4 (15.4)	0 (0.0)	2
Danforth et al., 2018^43^	MDMA	12	6 (50.0)	0 (0.0)	2 (16.7)	1 (8.3)	0 (0.0)	2 (16.7)	1 (8.3)	2
Wolfson et al., 2020^44^	MDMA	18	15 (83.3)	1 (5.6)	0 (0.0)	0 (0.0)	1 (5.6)	0 (0.0)	1 (5.6)	2
Mitchell et al., 2021^45^^,^^b^	MDMA	89	69 (77.5)	2 (2.2)	0 (0.0)	7 (7.9)	3 (3.4)	8 (9.0)	0 (0.0)	1
Davis et al., 2021^47^^,^^a^	Psilocybin	24	22 (91.7)	1 (4.2)	0 (0.0)	1 (4.2)	0 (0.0)	0 (0.0)	0 (0.0)	2
Bogenshutz et al., 2022^14^^,^^c^	Psilocybin	95	75 (78.9)	5 (5.3)	16 (16.8)	0 (0.0)	1 (1.1)	0 (0.0)	0 (0.0)	1
Goodwin et al., 2022^8^^,^^a^	Psilocybin (COMP360)	233	215 (92.3)	5 (2.1)	0 (0.0)	12 (5.2)	0 (0.0)	0 (0.0)	1 (0.4)	1
D'Souza et al., 2022^48^	DMT	7	6 (85.7)	0 (0.0)	0 (0.0)	1 (14.3)	0 (0.0)	0 (0.0)	0 (0.0)	3
Sloshower et al., 2023^12^	Psilocybin	19	16 (84.2)	2 (10.5)	0 (0.0)	0 (0.0)	0 (0.0)	1 (5.3)	0 (0.0)	3
Schneier et al., 2023^51^	Psilocybin	12	9 (75.0)	0 (0.0)	0 (0.0)	3 (25.0)	0 (0.0)	0 (0.0)	0 (0.0)	3
Raison et al., 2023^52^^,^^b^	Psilocybin	101	93 (92.1)	3 (3.0)	16 (15.8)	0 (0.0)	0 (0.0)	6 (5.9)	0 (0.0)	1
Mitchell et al., 2023^50^	MDMA	104	69 (66.3)	8 (7.7)	28 (26.9)	11 (10.6)	3 (2.9)	13 (12.5)	0 (0.0)	1
Goodwin et al., 2023^53^^,^^a^^,^^b^	Psilocybin (COMP360)	19	15 (78.9)	2 (10.5)	0 (0.0)	0 (0.0)	0 (0.0)	0 (0.0)	0 (0.0)	3
Peck et al., 2023^54^	Psilocybin	10	9 (90.0)	0 (0.0)	1 (10.0)	0 (0.0)	0 (0.0)	0 (0.0)	0 (0.0)	3
Davis et al., 2023^57^	5-MeO-DMT + Ibogaine	86	75 (87.2)	1 (1.2)	9 (10.5)	2 (2.3)	3 (3.5)	5 (5.8)	0 (0.0)	3
Aaronson et al., 2023^55^^,^^b^	Psilocybin (COMP360)	15	12 (80.0)	0 (0.0)	0 (0.0)	0 (0.0)	0 (0.0)	0 (0.0)	3 (20.0)	3
Agrawal et al., 2024^15^	Psilocybin (COMP360)	30	24 (80.0)	3 (10.0)	1 (3.3)	2 (6.7)	0 (0.0)	0 (0.0)	0 (0.0)	3
*Studies after 2017, N = 18*	*Total*	*928*	*778 (83.8)*	*33 (3.6)*	*76 (8.2)*	*40 (4.3)*	*13 (1.4)*	*39 (4.2)*	*6 (0.6)*	
*Pooled studies, N = 25*		*1074*	*908 (84.5)*	*36 (3.4)*	*80 (7.4)*	*43 (4.0)*	*15 (1.4)*	*40 (3.7)*	*9 (0.8)*	

Abbreviations: MDMA, 3,4-methylenedioxy-methamphetamine; DMT, N,N-Dimethyltryptamine; 5-MeO-DMT, 5-methoxy-N, N-dimethyltryptamine; USA, United States of America.

Note: studies that involved sites from multiple countries were included in this table if >50% of patients were recruited from the USA.

a Authors were contacted to provide missing data.

b Paper contained incomplete ethnoracial demographic data.

c Sum greater than 100% as some participants selected multiple categories rather than identifying as “mixed”.

d Includes individuals identifying ethnically as Latinx or Hispanic regardless of the selected race category.

e Quality Rating Scheme for Studies and Other Evidence: 1 = Properly powered and conducted randomized clinical trial; systematic review with meta-analysis. 2 = Well-designed controlled trial without randomization; prospective comparative cohort trial. 3 = Case-control studies; retrospective cohort study. 4 = Case series with or without intervention; cross-sectional study. 5 = Opinion of respected authorities; case reports.

Comparisons from USA studies found significant differences in ethnoracial inclusion between studies published before and after December 31, 2017. Earlier studies had smaller proportions of Black, Asian, Latinx, and Mixed-Race participants, and greater proportions of White and Other participants, suggesting some progress toward diversifying USA study samples. Consistent with these findings, proportions of White vs. non-White participants between USA studies published before and after December 31, 2017 showed notable differences.

Quality ratings of USA studies (Table 3) found that studies published before 2018 were of comparable quality with a median rating of 2 (i.e., well-designed controlled trial without randomization; prospective comparative cohort trial) compared to studies published since 2018, which also had a median rating of 2, with no significant difference (U = 54., p = 0.58.).

## Discussion

In the pursuit of innovative treatment models, interest in psychedelic therapies has robustly increased over the past two decades,^59^ and a growing number of clinical trials targeting various substance use and psychiatric disorders are currently underway.^58^ In this review, the majority of participants, 1183/1393 (85.0%), in all included studies (n = 39), identified as non-Hispanic White and there were very low rates of participation among Black, Asian, Latinx, Native/indigenous peoples (Table 1). Similar findings were present in a sub-analysis of studies conducted in the USA with 908/1074 (84.5%) of participants identified as White (Table 3). There has been minimal change in participation rates in studies published after 2017 when compared to studies published before 2018 overall. However, data from studies conducted in the USA did show differences in study sample demographics between studies published before and after 2018, with smaller proportions of White and greater proportions of minority participants in more recent studies. While notable, observed changes in diversity were small in absolute terms. Non-Hispanic White people account for 57.8% of the population in the USA per 2020 Census reporting,^60^ but remain over-represented in psychedelic clinical trials. Similarly, people identifying as Hispanic or Black, the second and third most prevalent ethnoracial groups in the USA, remain under-represented despite respective population prevalences of 18.7% and 12.1%. These are important findings given the increasing size of modern psychedelic trials and the larger breadth of scientific exploration of these agents as treatments in recent years (Table 2). The growing level of scientific curiosity in these treatments is made evident by the sharp increase, by a factor of 6.25, in the number of participants in included clinical trials from the USA after 2017 (Table 3).

Michaels et al.,^20^ previously reviewed ethnoracial inclusion rates in 16 studies published prior to 2018, which are also included in this review. In these studies, non-Hispanic Whites made up 232/282 (82.3%) of study participants with low rates of participation for ethnoracial minorities. In this review we found that non-Hispanic Whites remain the majority participant group, 951/1111 (85.6%), in studies published after 2017 (n = 23) and ethnoracial minority participation is again disappointingly low. Active attempts to diversify psychedelic research are likely responsible for the greater proportions of Black, Latinx, Mixed-Race and Asian participants in USA conducted studies after 2017. One study^32^ from 2013 accounted for 12/26 (46.2%) of all Indigenous participants and solely included aboriginal people in British Columbia, Canada. It is unclear why the proportion of indigenous participants has not substantially changed thereafter. Relations between non-white Indigenous and non-native White peoples have been historically turbulent worldwide and likely provide the basis for inclusion disparities in clinical research, but this question warrants further exploration. Studies differed in methodological approaches to collecting ethnic and racial identifiers, and many included studies did not separate ethnicity from racial identity at all. We acknowledge the nuances of ethnic and racial identity with emphasis on individual differences in self-identification as “Hispanic.” Though, if all participants from studies conducted in Spain, n = 6, and Mexico, n = 30, were categorized as Latinx, our main finding of primarily White participant inclusion would not be greatly changed. This would, however, increase the proportion of Latinx participants in included trials before 2018 substantially.

Despite increasing public and research interest and the conduct of larger and more rigorous trials, data indicate continued underrepresentation of ethnoracial minoritized populations six years after the previous review. While our results are striking it is important to keep in focus the ubiquity of diversity disparities in clinical trials across disciplines and specialties. Knepper and McLeod described a pervasive pattern of racial homogeneity of participants in clinical trials leading to FDA drug approval for treatments targeting heart disease, various cancers, and central nervous system disorders (41% of all drug approvals) between 1997 and 2014.^61^ Over this period the White participation rate in these studies ranged from 92% to 86% while Whites made up 72.7% to 62.2% of the population in the USA. Inadequate participant diversity is not unique to psychedelic clinical trials and in many ways clinical trials for common conditions fall short in this regard. It is also important to note that the included racial categories are quite broad and make comprehensive comparisons (such as rates of inclusion of South Asians vs Pacific Islanders) impossible. Precise collection of these demographic data in future studies may better assist in the identification and elucidation of disparities in ethnoracial inclusion rates.

The millennia-long use of psychedelic substances by indigenous non-White populations provides a crucial ethical perspective on the importance of diversifying psychedelic clinical trials. Psilocybin may be the most discussed psychedelic in both clinical and non-clinical contexts and to limit its investigation to primarily educated, non-indigent, White participants risks betraying the historical context of its use and neglects its potential to alleviate suffering for individuals of various ethnoracial backgrounds, many of whom face notable health disparities.^62^ Additionally, punitive and discriminatory drug policies within the USA have historically disenfranchised racial and ethnic minoritized populations. A social justice framework is therefore relevant to the methodological development of psychedelic trials to address these inequities which retrospectively cannot be corrected but can perhaps be remedied going forward through proactive consideration and action.

Since the publication of the previous review many landmark studies of psychedelic treatments have been published including two Phase III trials of MDMA for PTSD^45^^,^^50^ and the largest RCT to date of psilocybin.^8^ However, the present review indicates an ongoing diversity problem in psychedelic clinical trials. Notably, racial minorities face unique mental health challenges in the context of race-based trauma^63^ and discrimination^64^^,^^65^ perpetuated within negative societal constructs and embedded on a systems level within the USA. In these contexts, racial minorities may experience more severe, persistent psychological symptoms or functional impairment than their White counterparts,^64^ which can in turn be exacerbated by disparities in mental health care access.^66^^,^^67^ A recent survey-based study of 313 participants who identified as Black, Indigenous, or People of Color (BIPOC) in North America retrospectively assessed changes in mental health 1 month before and 1 month after ingestion of LSD, psilocybin, or MDMA.^68^ Significant (p < 0.001) reduction of symptoms of traumatic stress after psychedelic experiences with all three psychedelics was found (Cohen's d = −0.45). However, nationally representative survey data suggest ethnoracial background as a key moderator of mental health outcomes of naturalistic psychedelic use, with non-White individuals exhibiting lesser benefits on average, which may in turn be related to systemic challenges regularly faced among minoritized communities.^23^^,^^69^

Black people are more likely to have past-year or past-month hallucinogen use when compared to White people according to a retrospective analysis of data from the 2015–2019 National Survey on Drug Use and Health.^70^ Furthermore, Black, Hispanic, and Asian people are more likely to have lifetime use of MDMA compared to White people. In a pooled analysis of Phase II and Phase III trials testing the efficacy of MDMA-assisted therapy as a treatment for PTSD, no significant difference was found across ethnoracial groups.^71^ A recent survey-based study by Carter et al.^72^ found that Black Americans had more positive views of psychedelic assisted therapy than White Americans, and those with a greater baseline severity of depression or PTSD symptoms had greater interest in these treatments. These data reveal both a willingness to engage with psychedelic drugs, and potential for unique therapeutic benefits from psychedelic treatments in minoritized populations. Taken together, the evidence indicates both the compelling potential of psychedelic-assisted treatments to help mitigate existing mental health disparities among minoritized communities, while also calling into question the ability of any individually-focused mental health intervention to successfully ameliorate the systemic harms of social inequity, including criminal justice disparities, generational wealth inequality, and inaccessibility of adequate healthcare, housing, food, and education.

Within the USA, our society is structured in an ethnoracial hierarchy that historically has prioritized the needs and perspectives of White people over those of minoritized populations.^73^^,^^74^ The history of unethical research and experimentation on non-White individuals, mainly Black people, in the US has been well documented,^75^ and cannot be underestimated as a driver for participatory reluctance towards clinical research. More specifically, psychedelic research has historically exploited minoritized populations, including racial minorities and incarcerated persons, as far back as the 1950s and throughout the first wave of psychedelic research.^76^ Now that psychedelic science is again expanding, we must not repeat the regrettable mistakes of previous generations of researchers and must consciously diversify psychedelic clinical trials.

Race and ethnic-based discrimination from within the US healthcare system has been shown to negatively impact interactions and participation with, as well as perspectives of, the system itself while yielding poorer health outcomes within minoritized populations.^77^^,^^78^ From these findings one can infer contributions to the disparity in participation rates of non-White individuals in psychedelic studies. It is within this context of systemic oppression and ethnoracial-based inequities that approaches to increasing diversity and inclusion of minoritized populations in clinical research must operate. Furthermore, America's colonial history includes the genocides of Indigenous populations, the centuries-long enslavement of Black peoples, and discriminatory immigration policies targeting various East Asian, Southeast Asian, Latinx, and Arab groups. Through this lens of systemic oppression and discrimination, ostracism and othering, it may be difficult for members of minoritized ethnoracial groups to see the ways in which participation in clinical research could yield benefits specific to their communities; thus, lending to an understandably cynical view of White-majority institutions and White-led research endeavors. A refusal to acknowledge or consider these historical perspectives and their influence on the present state of diversity, inclusion, and equity in psychedelic research, and in clinical research in general, will undermine any efforts towards meaningful change.

Disparities in inclusion and access to psychedelic treatments and possible solutions have been discussed previously^79^^,^^80^ and detailed strategies for diversifying clinical and translational research in general have also been outlined.^81^ In the previous review,^20^ recruitment methods were discussed as potential barriers to inclusion of minoritized populations in these studies. The authors of this review reiterate these sentiments and enthusiastically call all to action.

Targeted efforts to understand the perspectives of ethnoracial minoritized populations regarding the use of psychedelic treatments in both clinical and non-clinical environments, particularly within catchment areas relevant to research centers, are paramount. It is likely true that cultural factors preclude willful involvement in these studies given the relational history of minoritized populations and White people in the USA as mentioned previously. The majority of psychiatrists,^82^ physicians and physician-scientists,^83^ and clinical researchers^84^ in the USA are White. It would be prudent to increase the participation of minoritized individuals as providers, researchers, and clinicians to increase relatability, cultural competency, and trust within the clinical or research encounter and beyond. This may be operationalized by implementing directives at legislative, institutional, and societal levels such as robust government funding for community-level initiatives, government-led initiatives, institution-sponsored educational pipeline programs, and public marketing campaigns aimed at increasing the involvement of minoritized populations with clinical systems and decreasing racial and ethnic stereotypes.

Participatory inclusion of cultural centers (places of worship, non-profit and community organizations, libraries, barber shops or hair salons, food establishments), relevant to ethnoracial communities, in these efforts may have a durable and robust impact by providing culturally sensitive perspectives and decreasing perceived stigma and bias towards clinical research and health care from within these communities. Collaborative community-based approaches aimed at engaging community members, from the beginning to the end of the research process, can help empower individuals and provide them with insight, knowledge, and results that are culturally relevant and meaningful.

While the executed literature review was thorough it is possible that some relevant studies were not captured and included. In addition, geographic and individual variations in the racial and ethnic identification and categorization of participants may contribute to difficulties in interpretation of these data. Methodological differences in the collection of race and ethnicity data also limit interpretation though the broad picture of White dominance in participant inclusion is clear. The use of broad racial categories without distinct subgroups disallows the precise capturing of diversity disparities and though effortful, if collected, would represent an earnest attempt at equitable methodological study design. We only included treatments involving classic psychedelics, MDMA, and ibogaine given the level of investigational interest in these compounds. Continued exploration of participant diversity rates within the full scope of clinical trials investigating all psychedelic and psychedelic-like molecules, as well as other new pharmacotherapies, will hopefully shed further light on means of increasing clinical trial diversity and ensuring equitable treatment access in the future.

In conclusion, the challenge of addressing long-standing structural discrimination and related contributions to disparities in ethnoracial minority participation in clinical trials is well understood. It appears that some progress towards diversity has been made within psychedelic treatment studies, particularly in the USA, but ethnoracial minority participation rates remain embarrassingly low despite an increased societal awareness of and focus on diversity, inclusion, and equity. It follows that robust efforts to diversify these studies must be prioritized with a culturally sensitive emphasis on increasing inclusion of these groups in all levels of clinical research–not solely as study participants. The fact remains, it is of the utmost importance to include diverse populations in studies of psychedelic treatments to promote health equity and ensure generalizability of these exciting and promising data.

## Contributors

MEH and AGR both contributed to the literature search, figure and study design, data collection, analysis, interpretation, and manuscript writing including revisions. Both authors accessed and verified reported data.

## Data sharing statement

Both authors agree that data supporting our findings are present within this manuscript. We agree to make these data openly available upon request.

## Declaration of interests

AGR is a paid scientific advisor to Innerwell. AGR has received research funding from MicroDoz Therapy Inc., Mydecine Innovations Group Inc., Unlimited Sciences, the Council on Spiritual Practices, the Heffter Research Institute, NIH, and DoD. AGR has received funding support through the Johns Hopkins Center for Psychedelic and Consciousness Research provided by Tim Ferriss, Matt Mullenweg, Blake Mycoskie, Craig Nerenberg, and the Steven and Alexandra Cohen Foundation. MEH has received funding support from the American Psychiatric Association Foundation and the American Academy of Addiction Psychiatry. All other authors declare no competing interests.
